# Combined donor-recipient genotypes of leptin receptor and adiponectin gene polymorphisms affect the incidence of complications after renal transplantation

**DOI:** 10.1016/j.ymgmr.2020.100648

**Published:** 2020-09-12

**Authors:** Sonia Mota-Zamorano, Enrique Luna, Guadalupe Garcia-Pino, Luz M. González, Guillermo Gervasini

**Affiliations:** a Department of Medical and Surgical Therapeutics, Division of Pharmacology, Medical School, University of Extremadura, Badajoz, Spain; b Service of Nephrology, Badajoz University Hospital, Badajoz, Spain; c Service of Nephrology, Hospital de Zafra, Zafra, Spain

**Keywords:** Leptin, Adiponectin, Genetics, Donor, Renal transplant, Graft loss, Delayed graft function, Graft function, *ADIPOQ*, adiponectin gene, CI, 95% confidence interval, CKD, chronic kidney disease, DNA, deoxyribonucleic acid, DGF, delayed graft function, eGFR, glomerular filtration rate, *LEPR*, leptin receptor gene, OR, odds ratio, SNP, single-nucleotide polymorphism

## Abstract

**Background:**

We aimed to examine whether combined donor/recipient variants in the leptin receptor (*LEPR*) and adiponectin (*ADIPOQ*) genes may affect outcomes in renal transplantation.

**Methods:**

A total of 233 donors and their corresponding 307 recipients were genotyped for *LEPR* rs1805094, rs1137100 and rs1137101, and *ADIPOQ* rs1501299 and rs224176. Combined donor/recipient genetic scores were created to investigate associations with delayed graft function (DGF), graft loss and estimated glomerular filtration rate (eGFR).

**Results:**

Recipients whose donors carried variant alleles of *LEPR rs1137100* and rs1137101 had lower risk of DGF [OR = 0.48 (0.24–0.97), *p* = 0.040] and [OR = 0.47 (0.23–0.95), *p* = 0.035], respectively. In addition, rs1137101 also showed an inverse association with lower incidence of graft loss [OR = 0.44 (0.31–0.97), *p* = 0.040]. The analysis of genetic scores of donor/recipients showed that again rs1137101 was inversely associated with both outcomes: OR = 0.46 (0.23–0.92), *p* = 0.029 and OR = 0.45 (0.11–0.81), *p* = 0.009, respectively. With regard to graft function, the T-allele of *ADIPOQ* rs1501299 in the donor was related to higher eGFR values (75.26 ± 29.01 vs. 67.34 ± 25.39 ml/min for wild-type grafts, *p* = 0.012). Higher combined genetic scores in this same polymorphism were also associated with better function (78.33 ± 31.87 vs. 68.25 ± 24.32 ml/min, *p* = 0.018). Finally, eGFR values were similar between paired kidneys but they were different when comparing grafts with or without the rs1501299 T-variant (77.87 ± 26.50 vs. 69.27 ± 26.73 ml/min, *p* = 0.016).

**Conclusions:**

Our study has shown for the first time to our knowledge that variants in *LEPR* and *ADIPOQ* genes of the donors and/or their combination with those in the recipients may affect the outcome of renal transplantation.

## 1 Introduction

Chronic kidney disease (CKD) is a health problem that affects 10% of the world's population and whose incidence has increased significantly in the last three decades [1,2]. Renal transplantation is usually the best alternative for patients diagnosed with end-stage renal disease (ESRD), and a deep knowledge of the immune system is key for its management. In this regard, white adipose tissue, which controls energy balance and metabolism, has lately been regarded as an important immunoendocrine organ. Various adipocytokines are synthesized in this tissue, of which leptin and adiponectin are the most widely studied [3,4].

There is an accumulating body of evidence pointing to an important role of these two adipocytokines in renal injury. Thus, leptin has been claimed to participate significantly in renal dysfunction, as it plays an important role in the onset and progression of glomerular endothelial proliferation, vascular damage, increased production of collagen and mesangium cells hypertrophy [[5], [6], [7]]. In the same manner, in patients with established CKD, adiponectin levels have been shown to be related to the progression of both CKD and ESRD [[8], [9], [10]]. However, the precise mechanisms by which these cytokines exert their effects on the kidney are still unclear.

Others and we have reported that leptin and adiponectin can also be important in the incidence of complications after renal transplantation. This is true not only for their plasma concentrations but also for the presence of variants in related gene loci [[11], [12], [13], [14]].

Even though leptin and adiponectin concentrations and gene polymorphisms seem to play a significant role of in the processes leading to renal injury, it is somewhat surprising that there are no studies in renal transplantation that have assessed the effect of allelic variants of the donor in these genes. In the present work, we have aimed to determine whether functional, clinically relevant, common SNPs in the leptin receptor (*LEPR*) or adiponectin (*ADIPOQ*) genes of the donor and/or their combination with the recipients' genotypes are related to graft function or complications such as delayed graft function or graft loss.

## 2 Materials and methods

### 2.1 Study design

The study sample consisted of 233 Caucasian deceased donors and their 307 corresponding renal transplant recipients (there were procedures in which both kidneys from the donor were transplanted into two different recipients). All patients received a single kidney at the University Hospital of Badajoz (Southwest Spain), which is the reference center for renal transplant in the region. Transplant recipients were recruited from the Renal Transplant Unit after giving written consent for their participation. At that time, a 10-ml blood sample was drawn and stored at −80C until DNA purification (recipient samples were collected after transplantation). Clinical records of the patients were retrospectively reviewed to collect all relevant data. The study protocol was approved by the Ethics Committee of the University Hospital of Badajoz and it was carried out in accordance with the Declaration of Helsinki and its subsequent revisions. No organs of prisoners or homeless people were included in the study.

After the transplant, patients were treated with a tapering schedule of corticosteroids (500 mg IV methylprednisolone at the time of surgery, 125 mg intravenously the next day and then 20 mg of oral prednisone every day, until the dose was reduced to 5 mg daily at 2 months after transplantation); mycophenolate mofetil (2 g per day); and calcineurin inhibitors (cyclosporine or tacrolimus). Starting doses of cyclosporine and tacrolimus were 4–10 mg/kg and 0.1 mg/kg, respectively, distributed into two administrations. The first dose was administered orally at the end of the transplant procedure or IV (one third of the oral dose) in the perioperative period. Doses of the immunosuppressive drugs were subsequently adjusted according to blood concentrations measured by a standard enzyme immunoassay performed on a Cobas Mira Plus analyzer (Roche Diagnostics, Basel, Switzerland). Basiliximab was used for induction immunosuppression.

### 2.2 Genotype analysis

Blood samples were extracted from the patients and genomic DNA was isolated by a standard method of phenol/chloroform extraction. Donors' DNA was purified from previously frozen lymphocytes using DNeasy Blood & Tissue kits (Qiagen, Hilden, Germany). We identified five variants in the *LEPR* and *ADIPOQ* genes, namely *LEPR* Lys109Arg (rs1137100), Gln223Arg (rs1137101) and Lys656Asn (rs1805094) and *ADIPOQ* 45 T/G (rs2241766) and 276G/T (rs1501299) by real-time PCR (TaqMan® SNP Genotype Assays, Thermofisher, Rockford, Il, USA). These variants are known to affect the levels and/or function of these genes or be involved in pathologies related to renal transplantation [[15], [16], [17], [18]]. Previously sequenced samples (5% of the total number) were used as positive and negative controls to verify the results.

### 2.3 Clinical variables

We studied the putative involvement of the studied genetic variants on the risk of delayed graft function (DGF), defined as the need for dialysis within the first week after transplantation; graft loss, defined as the absence of kidney function due to irreversible graft injury requiring chronic dialysis and/or re-transplantation; and graft function, which was evaluated with the estimated glomerular filtration rate (eGFR) using the Cockroft-Gault formula.

### 2.4 Statistical analysis

Univariate analysis were carried out for all the categorical variables that could influence the studied outcomes by using Pearson's X^2^ or Fisher's exact tests, as appropriate. Odds ratio and 95% confidence intervals were then calculated for all cases. To assess differences between quantitative variables and genotype groups, the Kruskal-Wallis/ Mann-Whitney or ANOVA/T-student tests were used depending on the normality of the data and the number of groups analyzed. Binary and linear regression models were built to study the effect of genetics on the different outcomes. The models included clinical and demographic covariates of patients according to clinical criteria or to the results of univariate studies. The covariates used in each model are specified in the *Results* section.

The dominant model of inheritance, i.e. non-carriers vs. carriers, was used in the genetic association analyses with the aim of obtaining groups with a balanced sample size. In addition, a genetic score was obtained for each donor-recipient pair, which was the result of adding the number of variant alleles in the donor and the recipient for each selected polymorphism. For statistical purposes, a binary variable was obtained by grouping this genetic score into two groups: Group 0 with pairs carrying none or one variant allele and group 1, consisting of pairs with 2 or more allelic variants.

In order to compare the weight of the donor vs. recipient genetics on renal function, we analyzed a subgroup of 170 kidneys that came from 85 donors, i.e. kidney pairs that had the same genetic background. Wilcoxon signed-rank tests were utilized to determine putative differences in graft function between paired organs.

The statistical power was evaluated with a genetic model that measured the frequency for carriers of the allelic variants with an arbitrarily effect size set at 2.5 and a type I error of 0.05. With the reported incidence of the outcomes considered and the available sample size, the power to identify genotype-phenotype associations varied from 0.797 to 0.841 depending on the minor allele frequencies (Quanto Software v. 1.2.4, USC). Statistical analyses were performed with IBM SPSS Statistics package v.22 (IBM Corporation, Armonk, NY, USA).

## 3 Results

The proportion of men and women and the age of the participants was similar in both the recipient and donor groups (63.2 vs. 66.1% of males and 49.16 ± 13.51 and 48.55 ± 17.90 years of age, respectively, *p* > 0.05). The ages in the recipient group ranged from 18 to 80 years.

Table 1 shows these and other characteristics of the study population. The incidence of the measured outcomes, DGF and graft loss, was 27.0% and 22.1%, respectively.Among patients with graft loss, approximately 3% had primary non-function. Mean eGFR in the recipients one year after grafting was 70.97 ± 27.38 ml/min (Table 1).

**Table 1:** Clinical and demographic parameters of the study population. Mean ± standard deviation values or number and percentages are shown. BMI, body mass index; CV, cardiovascular; DM, diabetes mellitus; HCV, Hepatitis C virus; eGFR, estimated glomerular filtration rate one year after transplant.

Parameter	
Age of recipient (yrs)	49.16 ± 13.51
Age of donor (yrs)	48.55 ± 17.9
Recipient males (%)	194 (63.2)
Donor males (%)	154 (66.1)
Time on dialysis (yrs)	4.32 ± 4.00
CV events in recipients	38 (12.4)
CV events in donors	26 (11.2)
BMI of recipients	27.75 ± 5.16
BMI of donors Hypertension in recipients	29.00 ± 7.28 241 (78.5)
Hypertension in donors	75 (32.2)
DM in recipients	32 (10.4)
DM of donor	24 (10.3)
Hyperlipidemia in recipients	97 (31.6)
Hyperlipidemia in donors	27 (11.6)
Smoking in recipients	60 (19.5)
Native kidney diseases	
Glomerulonephritis	111 (36.2)
Polycystic kidney disease	52 (16.9)
Chronic interstitial nephritis	41 (13.3)
Other	39 (12.7)
Undetermined	64 (20.8)
HLA mistmaches	
1–3	195 (63.5)
4–5	112 (36.5)
Delayed graft function	83 (27.0)
Acute rejection	48 (15.6)
Graft loss	68 (22.1)
Cold ischemia time (hrs)	17.23 ± 15.61
Revascularization time (hrs)	61.43 ± 20.67
Cyclosporine	42 (13.7)
Tacrolimus	265 (86.3)
anti Il-2 receptor antibodies	157 (51.1)
Mycophenolate	286 (93.2)
HCV infection	19 (6.2)
eGFR (ml/min)	70.97 ± 27.38

### 3.1 Association of donor polymorphisms with the outcomes in renal transplant recipients

Donor genotypes and allelic frequencies of the five leptin receptor and adiponectin SNPs included in the study in both donors and recipients are shown in Table 2.

**Table 2:** Genotypic and allelic frequencies observed in the population of study. N, number of subjects; MAF, minor allele frequency; HWE, Hardy-Weinberg equilibrium.

Polymorphism				Recipients				Donors	
		N	%	MAF	HWEp	N	%	MAF	HWE-p
*LEPR rs1805094*	GG	203	66.1	0.182	1	153	65.7	0.187	1
	GC	96	31.3			73	31.3		
	CC	8	2.6			7	3.0		
*LEPR rs1137100*	AA	172	56.6	0.247	0.888	130	56.0	0.25	0.920
	AG	114	37.5			88	38.0		
	GG	18	5.9			14	6.0		
*LEPR rs1137101*	AA	101	33.1	0.418	1	73	31.6	0.433	1
	AG	153	50.2			116	50.2		
	GG	51	16.7			42	18.2		
*ADIPOQ rs1501299*	GG	156	50.8	0.293	1	118	50.9	0.297	0.863
	GT	122	39.7			90	38.8		
	TT	29	9.4			24	10.3		
*ADIPOQ rs2241766*	TT	209	69.4	0.166	0.888	162	69.8	0.179	0.632
	TG	84	27.9			57	24.6		
	GG	8	2.7			13	5.6		

Genotyping was successful in 99.3% and 99.6% of recipients and donors samples, respectively. Minor allele frequencies range from 0.166 to 0.418 in recipients and from 0.179 to 0.433 in donors. There were no significant differences in the genotype frequencies of donors and recipients (X [2]-p > 0.05 for all SNPs). None of the identified polymorphisms showed significant deviations from the Hardy-Weinberg equilibrium in the population of study.

Logistic regression analysis controlling for meaningful covariates were performed to identify associations between donor SNPs and clinical outcomes. Recipients with grafts from donors carrying the G variant allele of the *LEPR rs1137100* or *rs1137101* SNPs showed a decreased susceptibility for DGF [OR = 0.48 (0.24–0.97), p = 0.040 and OR = 0.47 (0.23–0.95), p = 0.035], respectively.Table 3 shows the results for all the studied polymorphisms and the covariates utilized to adjust the model. In addition, the same G variant allele of rs1137101 was associated with decreased risk of graft loss [OR = 0.44 (0.31–0.97), *p* = 0.040, after controlling for clinical and demographic covariates (Table 3). Supplementary Table S1 shows the analysis of the same associations for the recipients' genotypes. No relevant associations were observed.

**Table 3:** Association of *LEPR* and *ADIPOQ* SNPs in the donors with delayed graft function one year after transplant and with the incidence of graft loss. B, regression coefficient; SE, standard error; df, degrees of freedom; OR, odds ratio; CI, 95% confidence interval. Results for delayed graft function were adjusted by age, BMI, diabetes, HCV infection, cause of donor death,acute rejection, use of basiliximab, HLA mismatch, cold ischemia time and revascularization time.,. For the graft loss model the covariates utilized were age, BMI, HLA mismatch, HCV infection, delayed graft function, diabetes, hypertension, history of CV events, hyperlipidemia, use of mycophenolate, use of basiliximab and acute rejection.

	B	SE	Wald	OR	CI	p
*Delayed graft function*						
*LEPR* rs1805094	0.706	0.356	3.930	2.03	(0.99–4.07)	0.051
*LEPR* rs1137100	−0.732	0.357	4.213	0.48	(0.24–0.97)	0.040
*LEPR* rs1137101	−0.756	0.359	4.430	0.47	(0.23–0.95)	0.035
*ADIPOQ* rs1501299	−0.481	0.349	1.901	0.62	(0.31–1.23)	0.168
*ADIPOQ* rs2241766	−0.354	0.378	0.877	0.70	(0.33–1.47)	0.349

*Graft loss*						
*LEPR* rs1805094	0.467	0.363	1.612	1.97	(0.46–3.15)	0.197
*LEPR* rs1137100	−0.310	0.380	0.656	0.65	(0.32–1.49)	0.430
*LEPR* rs1137101	−0.782	0.375	4.391	0.44	(0.31–0.97)	0.040
*ADIPOQ* rs1501299	0.012	0.338	0.011	1.21	(0.38–2.16)	0.946
*ADIPOQ* rs2241766	−0.021	0.401	0.007	0.89	(0.35–2.02)	0.976

**Fig. 1:**
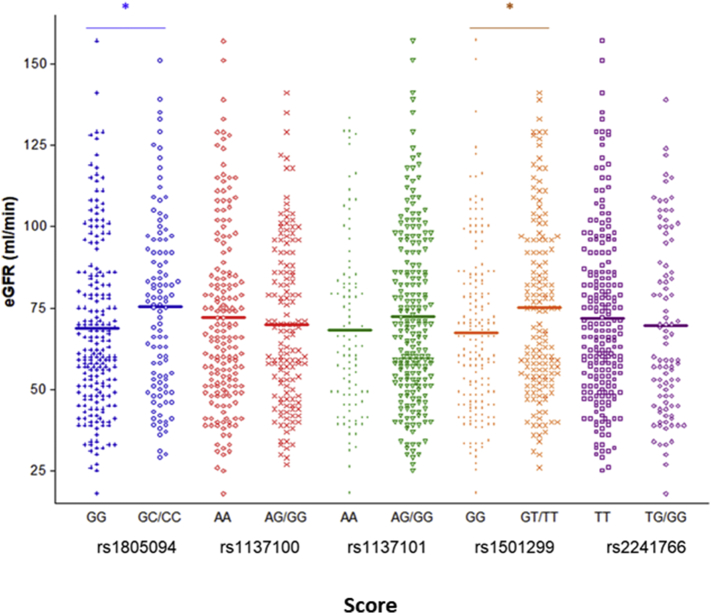
Distribution of estimated glomerular filtration rate (eGFR) values according to the genotypes of the five SNPs considered in the donor. Genetic association analyses were adjusted by age, sex, cold ischemia time, revascularization time, occurrence of DGF and acute rejection, hyperlipidemia, diabetes and history of CV events. *p < 0.05.

Finally, recipients with grafts from donors who carried the variant allele of two SNPs, rs1805094 and rs1501299, had higher eGFR values one year after transplant than patients with grafts harboring wild-type homozygous genotypes. Mean values for variant vs. wild-type genotypes were 75.36 ± 27.33 vs. 68.70 ± 27.18 ml/min (*p* = 0.033) and 75.26 ± 29.01 vs. 67.34 ± 25.39 ml/min (p = 0.012) for rs1805094 and rs1501299, respectively (Fig. 1).

### 3.2 Association of combined donor-recipient genotypes with clinical outcomes

Next, we examined whether the combination of donor and recipient genetics had any impact on the measured clinical outcomes. For this, we created genetic scores ranging from 0 to 4 depending on the number of variant alleles in each of the 307 donor-recipient pairs (Supplementary Table S2).

The analysis evidenced that *ADIPOQ* rs1501299 was shown to be associated with a lower risk of DGF when combining donor-recipient genetics [OR = 0.36 (0.16–0.82), *p* = 0.014] (Table 4). The statistical significance of the association of *LEPR* rs1137101 with decreased DGF risk that was previously observed in donors increased when combined donor-recipient genetics were analyzed: OR for high vs. low scores was 0.46 (0.23–0.92), *p* = 0.029 (Table 4).

**Table 4:** Association of donor-recipient genetics with delayed graft function (DGF). The low score group consisted of donor-recipient pairs carrying 0 or 1 variants in each considered locus, whilst the high score group are pairs with 2–4 variants. Ref., reference; OR, odds ratio; CI, 95% confidence interval. The covariates utilized to adjust the model are the same as those described in Table 3.

		No DGF		DGF			
	Score	N	%	N	%	OR (CI)	p-value
*LEPR* rs1805094	Low	196	86.3	65	81.3	Ref.	
	High	31	13.7	15	18.8	2.06 (0.86–4.92)	0.104
*LEPR* rs1137100	Low	169	74.8	56	72.7	Ref.	
	High	57	25.2	21	27.3	0.94 (0.42–2.07)	0.872
*LEPR* rs1137101	Low	88	38.9	42	53.8	Ref.	
	High	138	61.1	36	46.2	0.46 (0.23–0.92)	0.029
*ADIPOQ* rs1501299	Low	147	64.8	59	74.7	Ref.	
	High	80	35.2	20	25.3	0.36(0.16–0.82)	0.014
*ADIPOQ* rs2241766	Low	188	84.3	64	84.2	Ref.	
	High	35	15.7	12	15.8	1.04 (0.39–2.76)	0.938

The same trend was observed with regard to graft loss for this SNP, i.e., pairs with higher scores in the rs1137101 locus showed decreased risk of graft loss when compared to the low-score group [OR = 0.45 (0.11–0.81), *p* = 0.009] (Table 5).

**Table 5:** Association of donor-recipient genetics with graft loss. The low score group consisted of donor-recipient pairs carrying 0 or 1 variants in each considered locus, whilst the high score group are pairs with 2–4 variants. Ref., reference; OR, odds ratio; CI, 95% confidence interval. The covariates utilized to adjust the model are the same as those described in Table 3.

		**No graft loss**		**Graft loss**			
	**Score**	**N**	**%**	**N**	**%**	**OR (CI)**	**p-value**
*LEPR* rs1805094	Low	206	84.4%	55	87.3%	Ref.	
	High	38	15.6%	8	12.7%	0.58 (0.19–1.80)	0.422
*LEPR* rs1137100	Low	177	72.8%	48	80.0%	Ref.	
	High	66	27.2%	12	20.0%	0.71 (0.22–1.73)	0.383
*LEPR* rs1137101	Low	98	40.2%	32	53.3%	Ref.	
	High	146	59.8%	28	46.7%	0.45 (0.11–0.81)	0.009
*ADIPOQ* rs1501299	Low	169	69.3%	37	59.7%	Ref.	
	High	75	30.7%	25	40.3%	1.75 (0.68–3.44)	0.198
*ADIPOQ* rs2241766	Low	204	84.6%	48	82.8%	Ref.	
	High	37	15.4%	10	17.2%	1.26 (0.60–3.02)	0.735

**Fig. 2:**
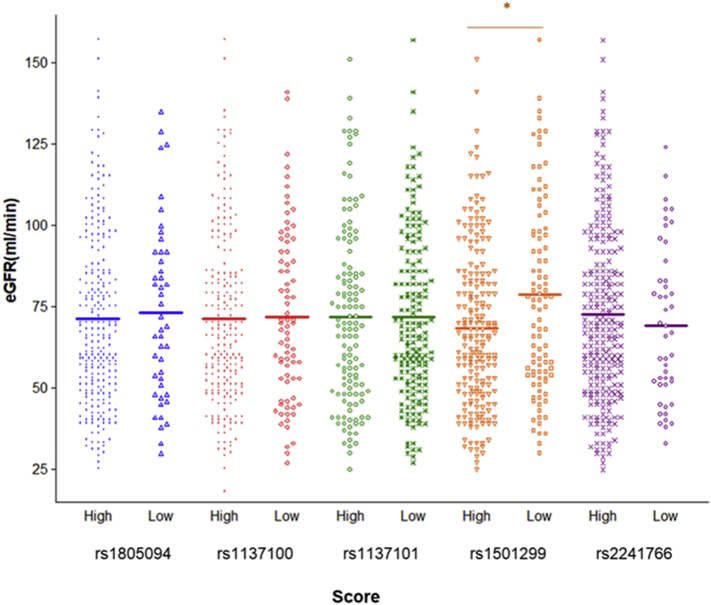
Distribution of estimated glomerular filtration rate (eGFR) values according to the genotypes of the five SNPs considered to the donor/recipient genetic score. Genetic association analyses were adjusted by age, sex, cold ischemia time, revascularization time, occurrence of DGF and acute rejection, hyperlipidemia, diabetes and history of CV events. *p < 0.05.

Finally, out of the two SNPs pointed out as modulators of eGFR in the analysis of donors genotypes, the rs1501299 retained significance when the genetics of the recipients were incorporated in the combined scores (Fig. 2). Patients with high scores in this SNP showed better graft function one year after the transplant than recipients in the low-score group did (78.33 ± 31.87 vs. 68.25 ± 24.32 ml/min, *p* = 0.018, Fig. 2).

### 3.3 Analysis of paired kidneys and graft function

**Fig. 3:**
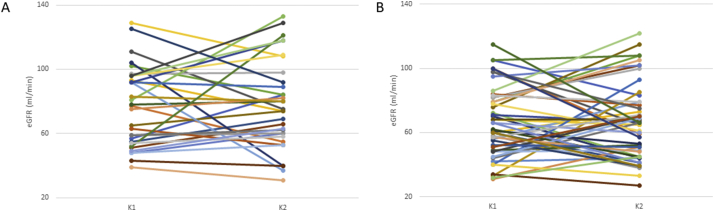
Estimated glomerular filtration rate (eGFR) for kidney pairs that carried (A) or not (B) the *ADIPOQ* rs1501299 T variant allele. K, kidney.

**Fig. 4:**
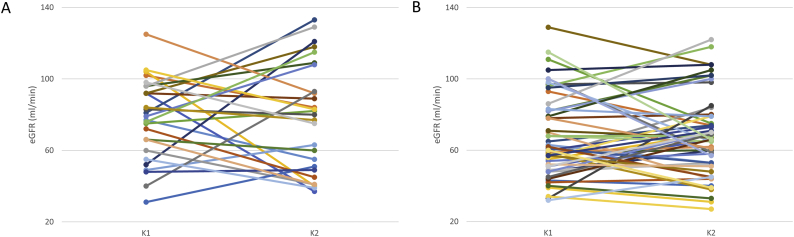
Estimated glomerular filtration rate (eGFR) for kidney pairs that carried (A) or not (B) the *LEPR* rs1805094 G variant allele. K, kidney.

In order to examine the weight of recipient vs. donor genetics with regard to graft function, the observed associations of rs1501299 and rs1805094 with eGFR were re-evaluated in a subgroup of 170 transplanted kidneys that were paired organs, i.e. from the same donor and therefore carrying the same genetic background. Fig. 3, Fig. 4 shows that there were no significant differences in the eGFR displayed by each of the kidneys in the paired transplants (paired *p*-values were 0.453, 0.904, 0.944 and 0.503 for the four groups depicted). However, kidney pairs carrying the rs1501299 variant genotypes (Fig. 3A) showed significantly higher eGFR values than wild-type pairs (80.0 ± 30.62 ml/min vs. 66.73 ± 22.59, *p* = 0.0018; Fig. 3B). In the same manner, donor-recipient pairs with the rs1805094 variant genotypes (Fig. 4A) had better graft function than noncarriers did (77.87 ± 26.50 vs. 69.27 ± 26.73 ml/min, *p* = 0.016, Fig. 4B).

## 4 Discussion

There are strong indications that leptin and adiponectin play a significant role in renal pathological processes. The results of the Olivetti Heart Study, a recent prospective trial with a long-term follow-up, supports a key causal role of leptin on kidney damage [7]. In the same manner, adiponectin levels have also been related to chronic kidney disease [9]. Moreover, a link to renal transplant has also been pointed out, as there are evidence of changes in the plasma concentrations of these adipocytokines after grafting [13,19]. We had previously reported that, besides circulating concentrations, genetic variability in these cytokines genes is also important for the outcome of renal transplantation [11,12]. However, to date there are no studies where the role of donor genetics, with the potential to be crucial for processes that occur locally in the graft, is evaluated.

Our findings show that two SNPs in the *LEPR* gene of the donor, rs1137100 and rs1137101 displayed an inverse association with the risk of DGF. Our group had already reported that the latter SNP, when found in the recipient, also showed an inverse association with the risk of DGF [12], which highlights the putative importance of this locus. Furthermore, when both the donor and recipient genetic variability was jointly analyzed in the logistic regression models, it was again rs1137101 the SNP that showed the strongest link to the complication, and, interestingly enough, with a highly increased statistical significance. In vitro studies have shown that the rs1137101 SNP is located in a receptor domain that is key for its activity and, as such, is likely to lead to lower receptor signaling, trafficking or surface expression [20], although the precise consequences are still unknown [17]. The acute kidney injury that results in DGF in the first days after grafting presents a marked inflammatory component [21]. Being leptin a predominantly pro-inflammatory cytokine [22], it is plausible that a diminished leptin receptor signaling in the graft, produced by the presence of rs1137101 in the donor, could result in less in situ inflammation and therefore lower risk of DGF. Should the variant be also present in the recipient, the effect on inflammatory processes would presumably be more noticeable. In line with our findings, Fonseca et al. have shown that leptin levels may be an independent predictor of DGF [23].

The same rs1137101 SNP in the donor was also the locus most significantly associated with graft loss in our cohort, which again puts the focus on this polymorphism. Remarkably, and mirroring the results for DGF, the statistical significance of the association was increased five-fold when analyzing donor and recipient's variants combined. Unlike in the case of DGF, however, we could not confirm an important role of this SNP in the recipient with regard to graft loss [12]. This study shared some of the participants with the present work, but donor DNA samples were unavailable, which could be the reason why no associations were found for rs1137101. The rationale for the involvement of this SNP in graft loss is similar to that described for DGF. Even though the specific functions of leptin locally in the kidney are yet to be fully understood, a lower receptor signaling would translate into a more favorable scenario regarding inflammation, and hence into an improved graft condition. Indeed, Moraes-Vieira et al. have shown in animal models how leptin deficiency reduces allograft reactivity, thereby contributing to increase allograft survival [24]. The incidence of both DGF and graft loss may be considered as high in our cohort; most likely, the growing use of expanded criteria donors and donation after cardiac death may be behind this observation.

When we looked into graft function, determined by eGFR one year after the transplant, two loci in the donor stood out as significant modulators, namely *LEPR* rs1805094 and *ADIPOQ* rs1501299. In this case, only the latter retained statistical significance when the donor-recipient genetic scores were analyzed, as recipients with higher scores for rs1501299 displayed 15% higher eGFR values than patients with low scores did. As we mentioned previously, there are no other studies that have analyzed the clinical consequences of adipocytokines SNPs in the donor. In fact, only a few reports have examined its clinical consequences when carried by renal transplant recipients [12,25,26]. In a previous study by our group in this setting [12], we already highlighted the need for more in vitro studies that can unequivocally characterize the consequences that the rs1501299 SNP produces in the protein. Indeed, there are reports that link the T-variant allele to increased [26] or decreased [27] adiponectin levels, and also to higher or lower risk of cardiovascular disease and diabetes, a controversy that has been discussed elsewhere [15,28]. In any case, adiponectin enhances anti-inflammatory cytokine synthesis, inhibits angiotensin II-induced inflammation and decreases albuminuria. Interestingly, these anti-inflammatory actions also take place locally in the kidney, as adiponectin receptors are present in renal cells [29]. Moreover, Tian et al. recently showed in rodents that adiponectin is a renoprotective agent that attenuates kidney injury and fibrosis [30]. Therefore, our results, pointing to a beneficial effect of the SNP (as it was associated with better graft function), would be in line with those reports linking the T-variant allele with increased adiponectin activity [26]. Another finding that supports this hypothesis is that rs1501299 was also observed to be associated with decreased risk of DGF in our cohort.

One last observation that suggests that donor genetics are crucial for processes that occur locally in the graft is that obtained from the study of paired organs. We showed that, when donor genetics were identical, there were no differences in the observed eGFR values, regardless of what particular rs1501299 genotype was carried by the recipient. Conversely, differences became apparent when kidney pairs carrying and not carrying the variant were compared.

This study has some limitations, the main of these being that leptin and adiponectin levels in the recipients were not available. Having these concentrations would likely have shed further light on the mechanisms underlying the reported associations. In any case, for these parameters to be significant, measurements should have been carried out before grafting and at least at several time-points in the first year after transplant. Being this a retrospective study, with many patients transplanted years ago, it was not possible to perform the determination of cytokines concentrations. Secondly, even though the sample size was enough to achieve a correct statistical power, the described findings could be better tested in larger cohorts. However, our hospital performs an average of 40 renal transplants per year, and therefore higher numbers than those presented herein are hard to achieve. In any case, our sample size is in the range of the great majority of genetic studies on renal transplant, particularly if they include genomic data from deceased donors. Another limitation is that we selected SNPs that have been consistently related with events of interest in renal transplantation, but, like in any candidate genes study, we cannot obviously rule out that other variants in these loci, or even related genes, such as leptin (*LEP*) or adiponectin receptors (*ADIPOR1*, *ADIPOR2*) may also play a significant role. In fact, in the light of the reported findings and other published data [31,32], these last genes also seem good candidates for further studies on renal transplantation. Finally, as it was discussed before, more in vitro studies that can unequivocally determine the biochemical consequences of *LEPR* and *ADIPOQ* genetic polymorphisms are needed.

### 4.1 Conclusions

In summary, our results indicate that variability in genes related to leptin and adiponectin may play an important role in the outcome of kidney transplantation. The inclusion of polymorphisms could therefore be useful in future predictive models of renal transplant outcomes, such as that reported by Irish et al. [33] or others that may be found online (https://optn.transplant.hrsa.gov/resources/allocation-calculators/kdpi-calculator/). We have shown, for the first time to our knowledge, that not only the recipient's genetics is relevant, but also that its combination with genetic variants that belong to the donor seems to deeply affect the clinical evolution of renal transplant recipients.

## Authorship

SMZ Participated in the performance of the research and the writing of the paper, EL and GGP participated in the recruitment of patients and the performance of the research, LMG participated in data analysis, GG participated in research design and in the writing of the paper.

## Funding

This work has been supported in part by grants PI15/00804 and PI18/00745 from 10.13039/501100004587Instituto de Salud Carlos III, Madrid (Spain) and grants IB16014 and GR18007 from 10.13039/501100014181Junta de Extremadura, Mérida (Spain) and 10.13039/501100008530Fondo Europeo de Desarrollo Regional (FEDER) “*Una manera de hacer Europa*”.

## Declaration of Competing Interest

The authors declare no conflicts of interest.

## Appendix A Supplementary data

Supplementary material Image 1
