# Supplementary material for: Combined donor-recipient genotypes of leptin receptor and adiponectin gene polymorphisms affect the incidence of complications after renal transplantation
Source: Mol Genet Metab Rep. 2020 Sep 12;25:100648. doi: 10.1016/j.ymgmr.2020.100648 (PMC7498839; doi:10.1016/j.ymgmr.2020.100648)
Supplement: Supplementary file 1 — Supplementary material [file mmc1.docx]

| Delayed graft function | | | |  |  |  |
| --- | --- | --- | --- | --- | --- | --- |
|  | B | SE | Wald | OR | CI | p |
|  |  |  |  |  |  |  |
| *rs1805094* | 0.171 | 0.363 | 0.222 | 1.19 | (0.58-2.42) | 0.638 |
| *rs1501299* | -0.270 | 0.349 | 0.597 | 0.76 | (0.39-1.51) | 0.440 |
| *rs2241766* | -0.000 | 0.375 | 0.000 | 1.00 | (0.48-2.08) | 0.999 |
| *rs1137100* | -0.058 | 0.349 | 0.028 | 1.06 | (0.53-2.10) | 0.868 |
| *rs1137101* | -0.563 | 0.372 | 2.285 | 0.57 | (0.28-1.18) | 0.131 |
| Graft loss | | | |  |  |  |
|  | B | SE | Wald | OR | CI | p |
| *rs1805094* | -0.563 | 0.396 | 2.021 | 0.57 | (0.26-1.24) | 0.155 |
| *rs1501299* | 0.472 | 0.348 | 1.842 | 1.60 | (0.81-3.17) | 0.175 |
| *rs2241766* | 0.039 | 0.384 | 0.011 | 1.04 | (0.49-2.21) | 0.918 |
| *rs1137100* | -0.441 | 0.357 | 1.532 | 0.64 | (0.32-1.29) | 0.216 |
| *rs1137101* | -0.232 | 0.356 | 0.426 | 0.79 | (0.39-1.59) | 0.514 |

Supplementary Table S1. Associations between leptin receptor and adiponectin polymorphisms in the recipients and renal transplant outcomes. Covariates used for the logistic regression models are the same as those used in the models depicted in table 3.

| **Polymorphism** | **Number of variant alleles** | **N** | **%** |
| --- | --- | --- | --- |
| *LEPR rs1805094* | 0 | 133 | 41.80 |
|  | 1 | 128 | 40.30 |
|  | 2 | 41 | 12.90 |
|  | 3 | 5 | 1.60 |
|  | 4 | 0 | 0 |
| *LEPR rs1137100* | 0 | 102 | 32.10 |
|  | 1 | 123 | 38.70 |
|  | 2 | 59 | 18.60 |
|  | 3 | 17 | 5.30 |
|  | 4 | 2 | 0.60 |
| *LEPR rs1137101* | 0 | 29 | 9.10 |
|  | 1 | 101 | 31.80 |
|  | 2 | 112 | 35.20 |
|  | 3 | 52 | 16.40 |
|  | 4 | 10 | 3.1 |
| *ADIPOQ rs1501299* | 0 | 81 | 25.50 |
|  | 1 | 125 | 39.30 |
|  | 2 | 76 | 23.90 |
|  | 3 | 22 | 6.90 |
|  | 4 | 2 | 0.60 |
| *ADIPOQ rs2241766* | 0 | 147 | 46.20 |
|  | 1 | 105 | 33.00 |
|  | 2 | 36 | 11.30 |
|  | 3 | 11 | 3.50 |
|  | 4 | 0 | 0 |

**Supplementary Table S2.** Number of variant alleles for each of the five loci considered in the 307 donor-recipient pairs. N, number of subjects.
